# Editorial: Experts' opinions in radiation and health: Emerging issues in the field

**DOI:** 10.3389/fpubh.2023.1168971

**Published:** 2023-03-17

**Authors:** Dariusz Leszczynski

**Affiliations:** ^1^Biological and Environmental Sciences, University of Helsinki, Helsinki, Finland; ^2^Frontiers, Lausanne, Switzerland

**Keywords:** EMF, 5G, human health, safety limits, environmental impact

Radiation, whether naturally occurring or emitted by man-made devices, is present in the environment and impacts human health. Radiation sources and exposures should be adequately considered in all public health policies aimed at the protection of the population's health in the increasingly technological world we live in. Currently, the use of wireless communication devices and networks, including the 5th generation of wireless technology (5G), is associated with an increase in health concerns in part of the population.

Research on the biological and health effects of exposure to man-made electromagnetic fields (EMF) has been conducted for decades. Over time a significant amount of knowledge on this topic has been obtained and this knowledge forms the basis for the current radiation safety guidelines. This rationale is used as justification for the claims that exposure to EMF in compliance with the current safety guidelines is safe for everybody, no matter a person's age, size, or health status.

However, numerous reviews of the published studies indicated a significant lack of knowledge on the effects of EMF exposures on human physiology (assessed in human volunteers), on the possible co-effects effects of EMF exposures with other man-made environmental pollutants, or on the possible effects on fauna and flora. Hence, a careful analysis of the to-date published science suggests that the current claims of the safety of the population from the radiation emitted by wireless technology are based on assumptions and do not sufficiently take into account the data from scientific experimental studies. Therefore, there are some legitimate scientific concerns about whether the current safety guidelines for wireless devices and networks are sufficiently protective for all populations.

As the Chief Editor of the Radiation and Health section of the Frontiers in Public Health journal, I initiated a dedicated Research Topic aimed at addressing the health risks associated with wireless radiation and understanding policies that are in place to protect humans and the environment from developing health issues and living a healthy life, as well as providing opinions on the validity and reliability of the current safety guidelines.

In their perspective article, Barnes and Freeman presented opinions on the limitations of the scientific evidence for the currently used EMF safety limits. They focused on the lack of studies aimed at establishing the biophysical mechanism of the observed biological effects from EMF exposures.

Barnes and Freeman pointed out that we know enough to conclude that the biological effects occur at EMF exposure levels well below the current safety limits:

“*It is now well established that biological systems respond to exposure to weak EM fields at energy levels well below the current safety guidelines which result in modification of their functionality without significant changes in temperature. These observations are adding to the debate over what should be done to protect the users of cellular telecommunications systems*.”

…and they point out the complexity of the evaluation of EMF science:

“*The lack of understanding and agreement in the scientific community on the physics, chemistry, and biology on the effects of exposure to low-level EM fields makes regulatory action nearly impossible at this point in time. The phenomena cross several scientific disciplines and require understanding and acceptance of how the linkages between electromagnetic fields at an atomic level due to electron and nuclear spins affect the chemistry of the human cell in ways that lead to problems with human health. Traversing these multiple disciplines not only requires credible data and rational theory on the cause-and effect relationships, but also requires expertise from multiple disciplines that typically do not intersect in the scientific community*.”

Barnes and Freeman have also pointed out that although lots of expensive research has been done it has not focused sufficiently on examining the biophysical mechanisms leading to the occurrence of the observed biological effects, including any non-thermal effects. The lack of a well-established credible biophysical mechanism is often used to dismiss the existence of the biological effects as mere artifacts.

Finally, Barnes and Freeman reminded us that:

“*In the US, most industries can be held liable for not pursuing research on the safety of their products*.”

…and that voluntary measures might be insufficient in the context of the unpredictability of human behavior. This, in the opinion of Barnes and Freeman, calls for better research to gain scientific justification for the safety limits.

De Vocht and Albers have reviewed all currently available review articles, in chronological order of publication, dealing with the very limited research on the possible effects of EMF frequencies used in the 5th generation (5G) of wireless technology and human health.

De Vocht's and Albers's justly concern was that:

“*Ideally, the peer-reviewed evidence synthesis literature should be free of […] non-scientific influences, but in practice, this is rarely, if ever, the case. To explore the narrative that formed the basis for the evaluation of health risks in the peer-reviewed scientific literature, the publications on the topic published during the first critical period of discussion are briefly reviewed and discussed*.”

By performing the review of the published studies in chronological order of publication date the authors looked at the development of the debate over the health hazard related to the deployment of 5G technologies. They observed that the early phase of the deployment of 5G correlated with publications by authors linked to the anti-5G movement. de Vocht and Albers suggested that this pattern of publications indicates efforts from the anti-5G (or more broadly anti-EMF) movement to control the narrative. The authors have somewhat compared this situation with the “*sugar-sweetened beverage research*” where the industry, in hope of controlling the narrative, was very active in the early stages of the research. Furthermore, de Vocht and Albers claimed that the early anti-5G movement's-narrative:

“…*relied mostly on reviews of lower methodological quality compared, with the subsequently published reviews by independent researchers and researchers with links to industry*.”

…and that is partly correct. Here, it is necessary to remember that there is a general agreement among EMF researchers that the majority of the EMF research, including the 5G research, is of poor methodological quality. Hence, any reviews are based on “*lower methodological quality studies*”. The problem is rather how these studies of lesser quality are being interpreted. And here, de Vocht and Albers pointed out, that 5G reviews themselves employed poor methodology when reviewing poor quality 5G studies.

It is also necessary to remember that the same poor quality scientific evidence is being used by the WHO, ICNIRP, IEEE-ICES, and numerous governmental agencies, like BfS or ARPANSA, to assure users of the safety of the current safety guidelines. Clearly, the poor quality of scientific studies on 5G does not give a clear answer to whether the guidelines are sufficient or insufficient to protect health.

However, de Vocht and Albers did not consider that the anti-EMF movement has grown up since the early 1980s and, clearly, has learned from the earlier mistakes. Namely, they have more evidence of possible negative health effects of EMFs emitted by wireless communications technologies and attempted to affect the deployment of the 5G before it fully happened.

It is also necessary to remember that the publication of reviews in established peer-reviewed journals is the only leverage that the anti-5G movement has if it wishes to influence the deployment of 5G technology and health-related policies. Other players, like the WHO, the ICNIRP, the IEEE-ICES, the GSM Association, and the Mobile and Wireless Forum all have direct access to governments and governmental agencies responsible for the deployment of the 5G and development of health policies. Hence, it is understandable that the health-concerned anti-5G movement needs to be active in publishing peer-reviewed articles if it wants its opinions to be noticed. However, their use of poor methodology when performing reviews is questionable.

McCredden et al. published an opinion claiming that the current safety guidelines are based on assumptions of safety and not on hard experimental data indicating safety. Unfortunately, their published opinion suffers from the same limitation often pointed out for such syntheses of the evidence, in that it is not sufficient to just list the numbers of studies showing effects and studies showing no effects. Indeed, to get a scientifically meaningful review it is also necessary to analyze the scientific quality of the studies to establish whether the conclusions claimed by the authors are indeed the correct ones.

McCredden et al. concluded that in their opinion the 5G evidence base for the millimeter-waves suggests that plausible health effects cannot be ruled out, and have urged to perform:

“…*sound scientific research, done carefully, using the best laboratory practices and sufficiently large samples to produce significant results, funded and overseen by trusted bodies with appropriate expertise*” and they called for “*precautionary actions to be taken by policymakers via the use of risk aversion strategies*.”

Levitt et al. in their perspective article suggest that:

“*There is enough evidence to indicate we may be damaging non-human species at ecosystem and biosphere levels across all taxa from rising background levels of anthropogenic non-ionizing electromagnetic fields (EMF) from 0 Hz to 300 GHz*.”

This opinion of certainty is convincingly based on what type of research has been done thus far:

“*Mice and rats have been the primary animal species used in research, but also rabbits, dogs, cats, chickens, pigs, non-human primates, amphibians, insects, nematodes, various microbes, yeast cells, plants, and others. Effects have been seen in all taxa, in various frequencies, intensities, and exposure parameters*.”

Levitt et al. concluded that:

“*Investigators have known since the early 1970's how EMF and RF couples with most animal species. Given our increasing ambient EMF levels, far more precise understanding of the molecular and cellular processes of electro and magneto-reception in non-human species is suddenly critical*.”

Finally, Levitt et al. have warned that

“*We may already be overwhelming some species' natural biological sensors that evolved over eons*.”

However, it is necessary to remember that while Levitt et al. is correct that various animal species were used in experiments, the actual EMF exposures used in these experiments were generally different from those these animals and plants experience in their natural environment. Therefore, while we know that these species can be affected by EMF exposures in principle, we need further research to determine whether the exposures they realistically experience in their environment have an impact on their physiology and their health. In this context, the call by Levitt et al. for developing/setting:

“*Long-term chronic low-level EMF exposure guidelines, which do not now exist, should be set accordingly for wildlife; mitigation techniques where possible should be developed; full environmental reviews should be conducted prior to the licensing/buildout of major new technologies like 5G; and environmental laws/regulations should be strictly enforced*.”

…will require extensive new research before environmental guidelines can be reliably determined. In the meantime, unfortunately, we have to rely on assumptions.

López et al. in their opinion article examined measurement protocols for EMF exposures in the human environment and called for optimization of measurement protocols.

López et al. point out that currently:

“…*scientific studies evaluate the possible effects of prolonged exposure to microwaves at the epidemiological level and in vitro or in vivo models. However, the use of different methodologies in radiation measurement processes and different configurations of exposure equipment, such as frequency, radiation power density, and exposure time; do not allow adequate comparison of results, which makes it difficult to draw conclusions*.”

López et al. criticize current ways of EMF exposures in experimental studies and suggest that:

“…*parameters such as frequency and modulation could be important when considering potential biological effects. Choosing intensity as the only determining parameter for the occurrence of effects is a reductionist conception*.”

The use of a reductionist approach might cause scientists to be unable to discover all, potentially meaningful, biological effects.

López et al. concluded that in order to achieve realistic exposures there is a need to develop such measurement systems that would:

“…*not only determine averaged field strengths but must be able to measure the peak amplitude over time and, consequently, the cumulative radiation*.”

An opinion article by Lin, notably a former member of the ICNIRP, states that while there has been significant progress in research the issues of the impact of EMF exposures on health remains unsettled:

“…*as for their impact on the radiation health and safety of humans who are unnecessarily subjected to various levels of RF exposure over prolonged durations or even over their lifetime, the jury is still out*.”

Lin clearly states that the evidence showing that EMF might cause cancer is growing:

“…*there are consistent indications from epidemiological studies and animal investigations that RF exposure is probably carcinogenic to humans*.”

Lin strongly argues that the recent animal studies have strengthened the earlier epidemiological evidence that EMF exposures might cause cancer. He considers it odd that safety guidelines setting groups, ICNIRP and IEEE-ICES, dismiss this animal evidence by nitpicking about methodological details:

“*While recognizing that the two recent large animal studies employed good-laboratory practices (GLP), and prolonged exposures of rats for their entire lifespan, the current revisions of safety protection guidelines and standards decided to nitpick with objections based on “chance differences” and exposed rat core-body temperatures of up to 1*°*C at 0.1 W/kg. Oddly, in choosing to do so, ICES (6) and ICNIRP (5) neglected the incongruity of suggesting a 1*°*C body-core temperature elevation as the putative cancer-causing agent*.”

“*If the groups that promulgate the safety protection recommendations assume what seems to be their stance regarding experimental results in rats by U.S. NTP/NIEHS that a whole-body temperature increase of 1*°*C causes cancer, then the safety or reduction factors of 50 recommended for the general population, or 10 for occupationally engaged working person would be borderline for the specified objective and practically worthless from the standpoint of protecting ‘safety'.”*

Lin doesn't call for the implementation of the precautionary principle but he considers that there is sufficient evidence to call for the implementation of ALARA where exposures to EMF should be as low as reasonably achievable:

“*The principle of ALARA—as low as reasonably achievable—ought to be adopted as a strategy for RF health and safety protection*.”

Finally, my opinion article (Leszczynski) calling for a debate on EMF and health, might be summarizing all of the concerns presented in the articles of this Research Topic when I am pointing out that, using exactly the same scientific evidence, different groups of scientists arrive at very diverse, often opposite, conclusions:

“*The diversity of interpretations of RF-EMF science reflects a broader problem of RF-EMF research. When the results of experimental studies are difficult to interpret, and the outcomes of studies are mostly ambiguous, it is up to individual scientists and groups of scientists to determine the significance of the results of such studies. Scientists who are more worried about the possible health effects will provide a different final evaluation of the ambiguous science than the scientists who are less worried about the possible effects*.”

I pointed out that just performing more research and more reviews is not the way forward because the research done thus far is not of good quality, while there also remain ethical and moral issues to resolve:

“*Despite the general agreement that the currently available scientific evidence is of poor quality and that there are significant gaps in the knowledge, this poor and inadequate scientific evidence is being used to claim that there is either no evidence of harm or that evidence of harm has been established. Such statements not only lack logic but also are morally and ethically questionable. If the scientific evidence used either to support claims of safety, or lack of it, is of poor scientific quality, then claims of safety, or lack of it, are unreliable because they lack solid support from quality scientific studies*.”

I consider that the poor quality of research might have an impact on the reliability of safety guidelines based on such poor-quality scientific data. For this reason, I call for a scientific debate where all players would meet and debate science with the aim of reaching a consensus opinion:

“*In conclusion, I recommend conveying a round-table debate that would assess the current status of the science on RF-EMF and health and would review the adequacy of the current safety guidelines. The round-table debate might not change the current status quo. However, in the current situation, where there are significant gaps in knowledge and current studies are widely regarded as of poor quality, it would be reassuring if scientists from this highly polarized research field would come together and engage in a meaningful debate*.”

Following the publication of my opinion article, I have contacted several scientists, from organizations that evaluate the scientific evidence concerning possible health effects from exposures to man-made wireless radiation-emitting devices and networks, to gauge their interest and willingness to participate in the proposed debate. The responses, in general, are a very disconcerting read.

The following scientists from the following organizations responded as follows:

- T. Samaras, EU-SCHEER—interested in principle
- J. Keshvari, IEEE-ICES—not interested
- L. Giuliani, ICEMS—interested
- Ron Melnick, ICBE-EMF—interested in testifying (presenting own opinion) but not in debate
- E. van Deventer, WHO—not interested because of the ongoing WHO evaluation of EMF research
- J. Schuz, IARC—fully relies on WHO and IARC opinions
- C. Sage, BioInitiative—not interested
- R. Croft, ICNIRP—not interested

In the context of opinion and perspective articles published in the Research Topic and in the context of the low interest in the proposed debate, it is important to ask:

*In a situation when scientific studies on EMF and health are of known and proven insufficient quality, what is the scientific, ethical, and moral responsibility of scientists when they claim that human health safety is already assured?*

## Author contributions

The author confirms being the sole contributor of this work and has approved it for publication.

